# *Mycobacterium chimaera* colonisation of heater–cooler units (HCU) in Western Australia, 2015: investigation of possible iatrogenic infection using whole genome sequencing

**DOI:** 10.2807/1560-7917.ES.2016.21.46.30396

**Published:** 2016-11-17

**Authors:** James Owen Robinson, Geoffrey Wallace Coombs, David John Speers, Terillee Keehner, Anthony David Keil, Victoria D’Abrera, Peter Boan, Stanley Pang

**Affiliations:** 1 Royal Perth Hospital, Perth, Australia; 2 Fiona Stanley Hospital, Perth Australia; 3 Pathwest Laboratory Medicine WA, Fiona Stanley Hospital Network, Perth, Australia; 4 Australian Collaborating Centre for Enterococcus and Staphylococcus Species (ACCESS) Typing and Research, School of Veterinary and Life Sciences, Murdoch University and School of Biomedical Sciences, Curtin University, Perth, Australia; 5 PathWest Laboratory Medicine WA, Hospital Avenue, Nedlands, Australia; 6 School of Medicine and Pharmacology, University of Western Australia, Crawley, Australia; 7 St John of God Pathology, Perth, Australia

**Keywords:** Mycobacterium Chimaera, Heater-cooler unit, endocarditis, Whole genome sequencing

## Abstract

Following the reported link between heater–cooler unit (HCU) colonisation with *Mycobacterium chimaera* and endocarditis, mycobacterial sampling of all HCUs in use in Western Australia was initiated from August 2015, revealing *M. chimaera* colonisation in 10 of 15 HCUs. After *M. chimaera* was isolated from a pleural biopsy from a cardiothoracic patient who may have been exposed to a colonised HCU, a whole genome sequencing investigation was performed involving 65 specimens from 15 HCUs across five hospitals to assess if this infection was related to the HCU. Genetic relatedness was found between the 10 HCU *M. chimaera* isolates from four hospitals. However the *M. chimaera* isolate from the cardiothoracic patient was not genetically related to the HCU *M. chimaera* isolates from that hospital, nor to the other HCU isolates, indicating that the HCUs were not the source of the infection in this patient.

## Introduction

*Mycobacterium chimaera* is a slow growing mycobacterium from the *Mycobacterium avium* complex. In 2004, *M. chimaera* was identified as a new species within the complex group [1] and has been associated with pulmonary infections, predominantly in immunosuppressed patients and in patients with pre-existing lung conditions such as chronic obstructive pulmonary disease and cystic fibrosis [2,3]. Since 2013, *M. chimaera* has been reported as a cause of prosthetic valve endocarditis, bloodstream and vascular graft infections in several countries in Europe and the United States (US) [4-10], linked to the colonisation of the heater–cooler units (HCUs) used during open heart surgery with postulated airborne transmission in the operating theatre [8,11].

In June 2015, an HCU manufacturer issued a warning, instructing hospitals to follow updated disinfection and maintenance procedures on HCUs and perform mycobacterial sampling. In response, private and public hospitals in Western Australia (WA) commenced mycobacterial sampling of their HCUs (all HCUs in WA have been purchased from the same manufacturer) in August 2015. In March 2016, a clinical isolate of *M. chimaera* was obtained from a cardiothoracic surgical patient in whom the surgery involved the use of one of the HCUs. This finding triggered an investigation to assess if this infection was related to the HCU.

## Case report and environmental sampling

A patient in their 50s underwent cardiothoracic surgery employing a HCU for cardiopulmonary bypass at Hospital 4 in December 2015. The surgery did not involve the implantation of prosthetic material. During this surgery, the HCU was placed as far as possible from the patient, with the exhaust towards the theatre exhaust vent and away from the patient.

Following the warning from the manufacturer, the water of all five HCUs at Hospital 4 was cultured and four of the five HCUs tested positive for *M. chimaera* in October 2015. All HCUs therefore underwent cleaning and disinfection following the manufacturer’s instruction and were then deemed safe for use, the risk of postponing surgery while waiting for culture results being greater than the potential residual infective risk. As part of the monthly testing protocol, the HCUs were again sampled in November 2015 and *M. chimaera* was again cultured from one of the five HCUs after a 53-day incubation, i.e. after the patient’s surgery. As the specific HCU used at the time of surgery was not recorded, it was not possible to conclude or exclude that the patient was exposed to a HCU colonised with *M. chimaera*. A process of recording the HCU used for each patient surgery has since been introduced.

Air sampling was also attempted from the operating theatres at Hospital 4, but the sampling plates were overgrown with other organisms such that interpretation of mycobacterial growth was not possible. Sampling from other hospital sources, such as potable water was not attempted.

One month after the operation, the patient developed bilateral pleural effusions and a pneumothorax with *Pseudomonas aeruginosa* isolated from the pleural fluid. During a 6-week course of piperacillin/tazobactam, the patient required four pleural drainage procedures, three for recurrent effusion and one for pneumothorax. One week after cessation of antibiotics, the patient redeveloped a pleural effusion and *P. aeruginosa* was again cultured. At this point the patient underwent decortication, and *M. chimaera* was cultured from a pleural biopsy. The patient was commenced on a combination of piperacillin/tazobactam, ciprofloxacin, azithromycin and ethambutol and slowly improved. Of note, the patient did not have signs and symptoms of disseminated *M. chimaera* infection. Mycobacterial blood cultures were not performed.

## Methods

Mycobacterial culture from HCUs was performed at the Western Australian mycobacterial reference laboratory. Mycobacteriology culture methods for water samples based on the 2010 Gastroenterological Society of Australia guidelines [12] and comparable with subsequent British [13] and European [14] guidelines for *M. chimaera* isolation were followed. Aliquots of 50 mL were centrifuged at 3,000 g for 20 min, the supernatant discarded and the remaining 1–2 mL decontaminated using n-acetyl-l-cysteine-sodium hydroxide/sodium citrate. Two BBL MGIT tubes (Mycobacteria Growth Indicator Tube, Becton Dickinson, Sparks, US) and two Gerloff’s egg slopes (with added nalidixic acid, vancomycin, amphotericin and polymyxin) were each inoculated with 0.5 mL of the processed sample and incubated for 8 weeks at 30 °C and 36 °C. Positive cultures were confirmed by acid fast staining, with subculturing on Middlebrook 7H11 plates for purity and identification. Single colony identification was performed by 16S rRNA gene sequencing.

The pleural biopsy was similarly cultured but without NaOH processing, with the clinical isolate initially identified on solid media at 30 °C after 21 days.

Whole genome sequencing (WGS), using aMiSeq platform (Illumina, San Diego, US), was performed on all HCU *M. chimaera* isolates, the patient isolate and a *M. chimaera* isolate from a non-cardiothoracic patient. *M. chimaera* strain MCIMRL6 (NCBI accession number: LJHN01000001), a clinical respiratory isolate, was used as the reference sequence [15]. The Illumina paired-end sequencing data, with an average of 70 × coverage depth, were analysed for genetic relatedness using the Nullarbor bioinformatic pipeline software [16] to identify single nucleotide polymorphisms (SNPs) in the core genome by comparison with the reference sequence. SNPs in recombination events were removed based on the method described by Feng et al. A maximum parsimony phylogenetic tree was constructed using MEGA (v7.0) [17].

## Results

Sixty-five specimens from 15 HCUs used in five WA hospitals were cultured for mycobacteria over a 12-month period from August 2015 to July 2016. The sampling pattern initially varied between hospitals but became more regular for all hospitals with HCUs over time as standardised testing intervals were established. Single mycobacterial isolates from 10 different HCUs from four hospitals, as well as the patient isolate and the second clinical isolate from a non-cardiothoracic patient were confirmed as *M. chimaera* by WGS. In addition, *M. intracellulare* and *M. gordonae* were also isolated from HCUs. The *M. chimaera* HCU isolates clustered into two groups, one from Hospital 4 and one from Hospitals 1–3. The two groups differed by 28 SNPs, with 2–17 SNP differences between isolates within a group. The isolate from the patient in Hospital 4 did not cluster with the Hospital 4 HCU isolates; it differed from them by at least 63 SNPs (Figure). Likewise, the non-cardiothoracic patient isolate did not cluster with the HCU isolates.

**Figure:**
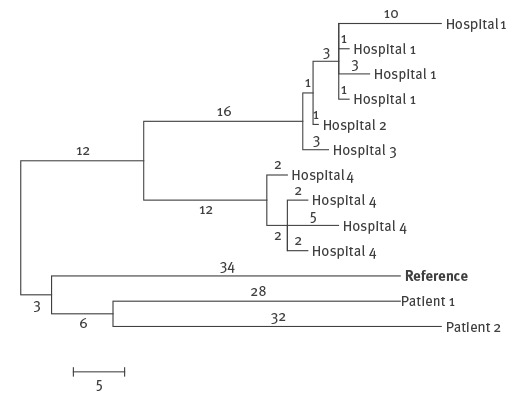
Genomic analysis of *Mycobacterium chimaera* strains grown from heater–cooler units from four hospitals, the cardiothoracic patient from Hospital 4 and a non-cardiothoracic patient, Western Australia, 2015–16 (n=12)

Patient 1: Hospital 4 cardiothoracic patient isolate, December 2015; patient 2: non-cardiothoracic patient isolate; reference: M chimaera strain MCIMRL6 (NCBI accession number: LJHN01000001).

Numbers indicate single nucleotide polymorphism differences.

## Discussion

An association of HCU colonisation with *M. chimaera* and subsequent deep tissue infections in cardio-pulmonary bypass patients has been reported in Europe and the US [5-7,11] but published molecular epidemiological information is scarce [8]. Our WGS investigation revealed frequent *M. chimaera* colonisation of HCUs across several hospitals in WA. The WGS results show that the HCU *M. chimaera* isolates in WA were genetically related as they all shared common SNPs, which is consistent with contamination from a common source. There was no transfer of HCUs between hospitals in WA to implicate a single hospital contamination event. The hospital tap water supply cannot be excluded as a source, but only sterile packaged water or filtered tap water is used in the filling and cleaning of HCUs in WA. Given that all the HCUs in WA were produced at the same manufacturing site, one hypothesis is the HCUs were contaminated during production at this site, as recently suggested by Haller et al. [6]. In their study they showed that isolates from cardiothoracic patients, HCUs and the manufacturing site were almost identical; however, their typing results have not been published. To examine this hypothesis further, systematic WGS of isolates collected from HCUs in multiple geographical locations is required. Notably, the two HCUs from the WA hospital that did not yield any positive cultures for *M. chimaera* were significantly older (more than 10 years) than the HCUs in Hospitals 1–4 and thus may have been manufactured before a possible contamination event at the manufacturing site.

The *M. chimaera* isolate from the cardiothoracic patient was not genetically related to the HCU *M. chimaera* isolates from that hospital, nor to the other HCU isolates, indicating that the HCUs were not the source of the infection in this patient. Although this finding is reassuring, the presence of multiple different strains in an individual specimen may not have been detected by our sampling method as only one colony was selected from the culture media for WGS. Furthermore, *M. chimaera* cases have been diagnosed up to five years after cardiovascular surgery [6] and therefore we may detect linked clinical cases into the future.

Interestingly, both patient isolates and the reference strain were from respiratory specimens but were not closely related to each other or to the HCU isolates. This would suggest heterogeneity in the environmental *M. chimaera* populations able to infect the respiratory tract of these patients and the HCUs. Due to the probability of contamination with *M. chimaera* at the overseas manufacturing site it is possible that the observed genetic differences between the patient and HCU isolates may simply reflect different *M. chimaera* populations in water sources in the two countries. It is currently unknown if different *M. chimaera* strains have different pathogenicity to cause infections of either prosthetic heart valves or the respiratory tract.

## Conclusion

Our study has demonstrated the usefulness of WGS in the analysis of a potential iatrogenic *M. chimaera* infection and shown that some HCUs used in WA are colonised with *M. chimaera*, as observed in countries on the northern hemisphere. As yet, no HCU-related infections have been identified in patients undergoing cardiopulmonary bypass procedures in WA. We must maintain a high level of suspicion in the population at risk while continuing regular disinfection and mycobacterial monitoring of our HCUs.
